# How kindness took a hold: A sociology of emotions, attachment and everyday enchantment

**DOI:** 10.1111/1468-4446.13128

**Published:** 2024-06-22

**Authors:** Julie Brownlie

**Affiliations:** ^1^ School of Social and Political Science Edinburgh University Edinburgh UK

**Keywords:** ambivalence, attachment, emotion, enchantment, kindness industry

## Abstract

How are we to understand the contemporary preoccupation—at least in many English‐speaking societies—with ‘random acts of kindness’ and the idea of kindness more generally? Should this be seen as a challenge to the logic of capitalism or reinforcing of it, an example of commodification of emotion within our everyday lives? By introducing and mapping the contours of an emergent ‘kindness industry’, placing emotion (and enchantment) at the heart of how attachment to the idea of kindness is theorised, and marshalling existing empirical research on contemporary framings of everyday kindness, I argue that there is a need for a critical sociological engagement with the ‘pro‐social’ that does justice to its profound ambivalence. In the case of contemporary kindness this involves understanding both the regulatory nature of the enchantment sold by a kindness industry *and* the problem‐solving potential of the enchantment of kindness in the everyday, where it both helps address contemporary feelings of hopelessness and shame and facilitates the possibility of making life materially liveable.

## 1 INTRODUCTION

According to internet lore, the injunction to ‘practice random kindness and senseless acts of beauty’ was coined in the early 1980s by American journalist and activist, Anne Herbert. ^1^ Herbert's own account emphasised its progressive origins, positioning it as a riposte to talk of ‘random violence and senseless acts of cruelty’ that was prevalent in Reagan's America.^2^

**FIGURE 1:**
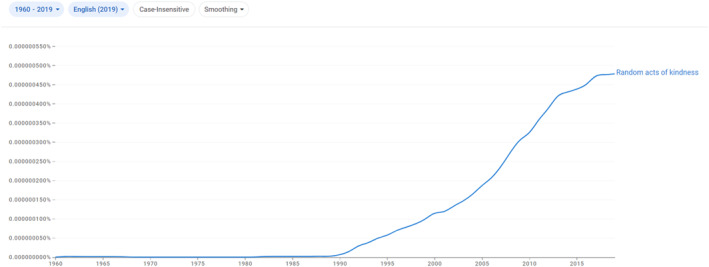
Frequency of references to ‘random acts of kindness’ in Google's English language corpus, 1960–2019. *Source*: http://books.google.com/ngrams.

The original formulation, then, referred to ‘acts’ only in the context of ‘senseless beauty’, a concept which apparently lacked cultural resonance and gradually fell away, allowing the idea of ‘random acts of kindness’ to emerge through a process of concatenation and truncation. Despite the contemporary ubiquity of the phrase, Google's corpora of books published in the English‐language shows that there were effectively zero instances of it before the late 1980s. Equally striking is how rapidly its use accelerated from the early 1990s onwards (see Figure 1).

In the period since, the idea of kindness—random or otherwise—has become a thoroughly public object or commodity, institutionalised through organisations, initiatives and publications. Although the word (and concept) has deep historical roots, and has come in and out of view across the centuries (Phillips & Taylor, 2009), there has been an extraordinary investment in the idea of kindness in recent decades, at least in the context of the UK, the USA, and some other parts of the (English‐speaking) world.^3^

How are we to understand this? In Herbert's original sense—as a spontaneous humanistic or solidaristic response to violence and other contemporary social ills? Or via a tradition in the social sciences of understanding moral practices (and associated emotions such as happiness or shame) through the lens of capitalism (Cabanas & Illouz, 2019; Davies, 2016), with the attendant risk that we are rendered as little more than cultural dupes for investing in such emotions and practices?

In this article, I focus instead on the ambivalences and contradictions of the idea of kindness (and of the enchantment that attaches to it). I introduce the notion of a ‘kindness industry’ but seek to go beyond its effects, to understand more fully how and why the idea of kindness currently comes to matter to people—even those who are critical of it. Put another way, I am interested in understanding why it is, to paraphrase Stevi Jackson, that even sociologists are kind.^4^

In this respect, my aims can be usefully compared with Cazenave's (2023) recent *Kindness Wars*, which dismisses contemporary investment in ideas of random acts of kindness (RAK) as producing ‘warm and fuzzy, puppy‐dog‐like’ books that inspire ‘the next generation of benevolent tree huggers’ and instead sees its mission as the ‘kick ass’ one of offering a ‘large, robust, and politically engaged conceptualization of kindness’, one where ‘arguments and actions for, or against, kindness *above* [my emphasis] the interpersonal level are inherently political’.

Contra Cazenave, I place the interpersonal at the *core* of the idea of kindness (including RAK)—both as it is deployed in everyday usage *and* as a proto‐political phenomenon, because of the profound social impact of imagined and actual everyday acts and interactions. This is an important point, given the scepticism about (or simple neglect of) the possibility of social change arising from people acting (*and feeling*) interpersonally rather than only as members of larger groups.

Cazenave starts his book by arguing that one of the advantages of kindness conceptually is its ‘everydayness’, yet one of the key ways it is understood in the contemporary world—as random—is summarily dismissed. From a position of wanting to avoid treating people as kind ‘robots’,^5^ I aim to take seriously what it is about this idea of randomness in particular that people connect to; and why they might be particularly drawn to it in the current moment.

In what follows, I show that ideas of kindness, and specifically RAK, have certainly been marketised—in part, through a sense of enchantment. Instead of being passively consumed, however, I argue that this sense of enchantment has taken a hold in contemporary times because it offers the possibility of ‘making a difference’ and hence of managing the emotional complexities of feeling hopeless while also practically making life bearable. Notions of enchantment are at the heart of attachment to contemporary idea of kindness, in other words, because they are a problem‐solving response to contemporary socio‐political upheaval, both materially and emotionally. Ideas of enchantment though are also a response to the problem *of* kindness and its ambivalences. In other words, framing kindness as enchanting is also a way of managing the difficult emotions it provokes, particularly feelings of vulnerability and shame.

I make the case, then, for understanding attachment to the enchantment of kindness as a more active process, an argument rooted in everyday sensemaking that echoes perspectives from interactionism and feminism, among others.

Drawing on an analysis of the kindness industry and of this everyday enchantment, in the context of an increasing sense of the world as individualising, rule‐following and instrumental, I conclude by making the case for ambivalence to have a more explicit part in social theoretical thinking about the ‘pro‐social’ in the context of contemporary capitalism.

## 2 A NOTE ON TERMINOLOGY

To be clear, I am not interested in offering a definition of kindness, but in the varying constellation of ideas, practices and emotions to which the word attaches. It is, then, never *just* an idea that we attach to: instead ‘the idea of kindness’ is a shorthand for the less catchy ‘the idea/practice/emotions that constitute the constellation that goes by the name of kindness’. This understanding also implicitly involves relationships, as it is taken as read that emotions, practices and ideas happen between people (or impact others), and also involve non‐human beings and objects. In other words, I offer here a particular sociological project: one which is interested in the work that the idea of kindness does, and in how and why we come to attach to this idea in some contemporary societies. This is a project that inserts emotions at the heart of theorising ambivalent attachment to kindness and, hence enchantment, and through doing so, aims to contribute to the wider sociological endeavour of not reducing the values associated with caring (broadly defined) wholly to value or to the logic of capital (Skeggs, 2014). A decade on from Skeggs' call in this journal for sociologists to understand that ‘what really matters to us is other people’ her argument, now inclusive of care beyond humans, remains core to the sociological project.

## 3 RAK AND THE KINDNESS INDUSTRY

Following Herbert's earlier intervention, in 1993, a small American publisher called Conari Press produced a book entitled ‘Random Acts of Kindness’ (Herbert & Pavel, 1993). In response to a wave of interest, in 1995, Conari's owner, Will Glennon, established a not‐for‐profit organisation called the Random Acts of Kindness Foundation. Over time, this came to act as the US delegate to the World Kindness Foundation—an international organisation, now with representation from some 30 countries.^6^ Through events such as ‘Random Acts of Kindness Day’ and ‘World Kindness Day’, these two organisations have played a key role in promoting kindness as a (nominally apolitical) normative agenda, simultaneously invoking, and contributing to, the reproduction of a global or world moral culture (Inglis, 2011).

**FIGURE 2:**
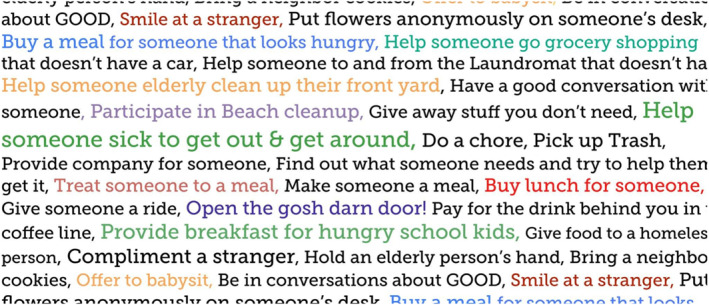
Ideas for kind acts for World Kindness Day. *Source*: https://www.kindnessevolution.org/.

There are now a large number of other organisations making up the kindness ecosphere, locally, nationally and internationally. A search of a non‐profit database in the US, for example, returns more than 600 organisations with kindness in the title, while a similar search in the UK identifies 27.^7^ In previous eras, kindness was talked of as primarily a character trait or relational quality (Pollock, 2011) but most of these organisations refer to kindness in terms of discrete (often ‘random’) transformative *acts* (see Figure 2).

KindSpring, for example, calls attention to evidence of a ‘global movement of kindness’ and keeps a running tally of ‘stories shared’.^8^ The website One Billion Acts of Kindness dispenses with the need to share stories and simply asks ‘Have an act of kindness to share? 1 CLICK = 1 ACT’, offering a bespoke app ‘to make sure your acts of kindness are counted’. (Its running total as of December 11, 2023 stood at 14,876,500).^9^

In the remainder of this section, I draw on searches of online content using simple search terms on Google, and specialist, comprehensive bibliographic tools for searches within academic and lay publishing. In doing so I expand on the place of RAK in the kindness industry, and explore some of this industry's key features, including ‘scientisation’^10^ (Green & Moran, 2021), professionalisation, commodification and politicisation.^11^

## 4 THE SCIENCE OF KINDNESS

Since the 1980s, the cultural preoccupation with kind ‘acts’ noted above has led to a focus on measurement. Being able to count or measure kindness and its effects has been core to it being treated ‘scientifically’ (Davies, 2016). For the last 15 years, at the heart of this new science of kindness, as was the case with happiness before it, has been research in positive psychology. Most of the organisational investment in kindness has been nominally other‐oriented—that is, in pursuit of a ‘kinder society’—but positive psychology's emphasis has also been on the wellbeing and happiness of individuals of performing kind acts and the notion that by *doing* good we *feel* good. This research is, in turn, increasingly referenced by the organisations mentioned above and in the process the goals of individual and global wellbeing become blurred.

The influence of positive psychology in the context of kindness, however, is relatively new. Academic publications on kindness prior to the 1980s, even though they were far fewer in number, were much more likely to be in the fields of philosophy and religious studies, with publications on kindness within sociology remaining relatively rare across the period as a whole. Analysis of journal outputs referencing kindness (based on Web of Science data) highlights a dramatic increase in kindness‐related outputs in the last 2 decades^12^ especially within psychology, health and neuroscience.

For positive psychologists, happiness and pro‐social behaviours are bidirectional: ‘there is robust evidence that “do good, feel good” and “feel good, do good” both apply’ (De Leeuw et al., 2023). Witnessing ‘moral beauty’ (Haidt, 2003), including on social media, it is claimed, leads to ‘elevation’—a desire to be a better person and to do good for others. Ko et al. (2019) take this one step further and suggest that ‘the ‘act’ element of an ‘act of kindness’ could be ‘optional’: in terms of ‘efficiently improving wellbeing’ it may be enough to *remember* acts of kindness. At the same time, there is also a growing reliance on neuroscientific research which again, as with previous research on happiness, highlights the role of oxytocin (a neurotransmitter and hormone) (MacFarlane, 2020, p. 1).^13^ Illouz (2019) maps a similar trajectory from psychology to brain chemistry in ‘the making of love into a science’.

Despite calls for more nuanced investigation of who is receiving and giving kindness and in which social contexts, and decades of feminist research cataloguing the costs for those doing good for others (Hochschild & Machung, 1989), there remains a strong focus on the pleasure for the ‘giver’ of giving. Positive psychology's influence has also shaped how kindness has become institutionalised in higher education, with an overlap often between academic centres, the kindness organisations described earlier and broader popular culture.^14^ These connections suggest the academy is both a driver and a beneficiary of the emerging kindness industry.

The science of kindness has in turn fuelled ‘kindness consultancy’. Part of a broader wellbeing ‘consultancy circuit’ (Davies, 2016), this supports a culture of kindness within schools, workplaces and other settings. The Kindness Corporation, for example, offers to certify employers as #WorkKind companies^15^ and accreditation of kindness is emerging, too, within the education sector.^16^

## 5 CAPITALISING ON KINDNESS

These developments align kindness with Davies's understanding of happiness as ‘an advantage’—claims that kinder people achieve more in their careers and enjoy better health turns kindness into a form of capital.^17^ Within the corporate world, kindness is not only increasingly seen as a driver of effective—and therefore profitable—relationships within workplaces but as a specific mechanism that can be deployed in support of branding activity, specifically through ‘RAK marketing’. The following offer from the British supermarket chain, ASDA is an example of this—and should be viewed in the context of its turnover, in 2022, of more than £20 billion.

Random Acts of Kindness Day® is on Thursday 17th February and to celebrate, ALL of Asda's 205 Cafés will be giving away five free hot drinks to customers at random throughout the day—that’s 1025 free drinks up for grabs!^18^

## 6 THE POLITICS OF KINDNESS

The industry and science of kindness outlined above promotes kindness as a politically neutral phenomenon, but needs to be seen in the context, since the 1990s, of a politicised ‘relational turn’ in thinking about the role of the state in many societies in the Global North. Against a backdrop of a shrinking welfare state, and a concomitant focus on civic and charity work, a number of think tanks, third sector organisations and politicians, from 2015 onwards, began to draw on the idea of kindness as a potential focus of public policy.^19^ Kindness has also begun to feature in political discourse as a form of ‘caring nationalism’ (Wood & Skeggs, 2020). Perhaps the best‐known example of this is Jacinda Ardern's New Zealand administration which repeatedly invoked the idea of kindness, including in response to the aftermath of the Christchurch shootings and the Covid‐19 pandemic. Ardern subsequently faced criticism from both the libertarian right (for the perceived absence of kindness in imposing COVID restrictions) and the left (for individualising systemic problems).

In summary, in the UK but also more widely, there has been an institutionalisation of the idea of kindness spanning politics, industry, science and markets—its core features of scientisation, professionalisation, commodification and politicisation familiar from a predecessor industry around happiness. Whether kindness *is* the new happiness or a reaction to its individualising qualities, there is certainly evidence that positive psychology, which underpinned the happiness industry, has been ‘bootstrapped’ on to contemporary understandings of kindness.

For those writing critically about happiness in recent years, this commodification is where the story has tended to stop. In the remainder of the article, however, I seek to understand why the idea of kindness might be invested in currently beyond it simply being sold to us as a way of ‘feeling good’. To do so involves developing a richer sociological understanding of attachment and enchantment, one with emotions at its heart.

## 7 THEORISING CONTEMPORARY INVESTMENT IN THE IDEA OF KINDNESS: FROM EMODITIES TO (AMBIVALENT) ATTACHMENT

As noted at the outset, the idea of kindness (and the emotions associated with it) can be viewed as part of a history of how moral and interpersonal relations are shaped by the needs of capitalism (Haskell, 1985; Hochschild, 2003; Zelizer, 2005).^20^ Fraser (2017) has argued this has culminated, in the context of financial capitalism, in the market undermining the social reproduction needed for it to survive, with the so called ‘crisis of care’ a consequence of this. Notions of enchantment are a core part of how this commodification, including of interpersonal relationships, can be understood. Given the extent to which kindness and happiness are intertwined, the happiness industry offers an obvious start point for thinking through some of these connections.

In 2016, William Davies published *The Happiness Industry*: *How Big Business and Government Sold Us Wellness*. Four years later, Cabanas and Illouz (2019) published a similarly titled book, *Manufacturing Happy Citizens*: *How Science and the Industry of Happiness Control Our Lives*. Both accounts focus on neoliberal capitalism and the management of emotional lives; and both are critical of attempts to measure happiness as a neutral fact and of the individualising nature of contemporary economic and wellbeing discourses.

As we have seen, many of the features Davies identifies in relation to happiness are relevant to kindness, not least because of the similar influence of positive psychology and the extension of the contemporary association of morality with happiness from the notion that a person who feels happy is a good person to the idea that a good person is a happy person.

Both books reflect on the taken‐for‐grantedness of the significance of happiness but also hint at its enchanting qualities—Davies through reference to its ‘magical’ quality or ‘luminosity’ and Cabanas and Illouz through the concept of ‘emodities’—something that promises ‘emotional transformation circulating and exchanged in a market’ (2019: 6). Cabanas and Illouz also investigate the cultural investment in happiness, exploring why it this, rather than say solidarity, which has come to play such a dominant role in advanced capitalist society. Their answer: happiness is ‘saturated with individualist values’. Consistent with Illouz's earlier work on consumer culture where, she argues, there can be no subjectivity ‘outside the compass of capitalism’^21^ (2018: 22), for Cabanas and Illouz (2019:216), we have become ‘psytizens’ controlled via the promotion of ‘continuous self‐optimisation’.

Davies does call for greater investigation of what individuals understand happiness to involve but his emphasis, too, remains on the political and economic determinants of an emerging happiness industry. In these accounts a sense of how lives are made sense of beyond the reach of such industries is scarce. To gain a fuller appreciation of this sense‐making in relation to kindness, to why and how kindness has taken a hold, I first turn to those who have written about attachment and then to those theorising enchantment, connecting both through a sociological lens on emotions.

## 8 ATTACHING TO THE IDEA OF KINDNESS

Though the language of attachment brings its own complications—like kindness, it is not an easily defined or singular idea—it offers a potentially compelling way to move beyond seeing the idea of kindness solely as an emodity that is passively consumed. In what follows, I touch briefly on what it means to attach in general then look at attachment through the lens of ideas and cultural objects, before turning to work on attachment as practice. It is possible to think of kindness as both a cultural object and a practice—and I give examples of both below—but it is the role of emotions in attaching to kindness that I primarily focus on, because understanding the contemporary mattering of kindness involves understanding how kindness *feels* in this moment as much as what it allows to happen in the everyday.

## 9 WHAT DOES IT MEAN TO ATTACH?

There is no one theory of attachment nor any one discipline that can lay claim to it. Traditionally, in psychology—and, to some extent, sociology—attachment relates to significant bonds (Redman, 2008). The concept has been further developed through Actor Network Theory (see Hennion, 2017 for a brief summary of this history) and cultural sociology (Heinich, 2021; Pomiès & Hennion, 2020). Sociological thinking, however, has also been influenced by work of cultural and literary theorists on attachment—most notably Berlant (2008), Ahmed (2010) and Felski (2020)—all of whom have offered slightly different readings. For some, attachment is their primary concept (Felski, 2020), for others a secondary one (McDonnell, 2023) but, broadly, it is understood as involving a sense of being ‘taken a hold of’. For Felski, it is a state of being both moved and tied to something. While her focus is art, she understands attachment as involving a sense of connection that is potentially physical, emotional, institutional or cultural but is, in essence, about coming to feel that something matters (Felski, 2020). She is clear, however, that this mattering is not purely emotional and applies to all sorts of relations including, crucially, those forged through ideas. Capitalism can shape these ideas, but it cannot, Felski suggests, completely explain why some ‘catch on’ (2020: 77).

How things come to matter can, of course, also be explored through the lens of morality (Abbott, 2019), yet the sociology of morality has not always paid close enough attention to *how* these relations of mattering, including those beyond the interpersonal, come about and how they are *felt*. Attachment's analytical edge is that it potentially allows for a detailed focus on both, but there are clearly also challenges of working with this concept: wide variability in how it is used; vagueness about what is actually meant by it; a restricted focus on aesthetic objects in some writing about it and, crucially, the fact that existing work does not always explicitly address *sociologically* the role of emotions. I aim to redress this by taking a sociology of emotions approach to attachment, seeing it as a socio‐historically embedded process that involves ideas, practices and emotions.

In the case of contemporary kindness, this means placing attachment in the context of socio‐economic and political upheaval. The distinctiveness of this contemporary, for some ‘late modern’, society has been examined through an emotions lens (Patulny & Olson, 2019). Barbalet (2019), for instance, has argued that neoliberal processes including globalisation and corporatisation have led to a focus on self (emotional) management. The less opportunity people feel they have to influence macro political economic processes, the more the range of emotions shrink so that ‘persons are likely to experience themselves as centres of emotion’ (174).^22^ Previously, individual emotions were transcended through, or contradicted by, institutions such as trade unions whereas now, Barbalet (2019)argues, relations with others are expressed ‘in terms of contractual obligation, interpersonal trust, or some other construction of ego emotion’. Barbalet sees this distinction between ego and other directed emotion reflected in Adam Smith's distinction between the ‘moral sentiments’ through which social order is preserved and ‘self love’.

Attachment to the idea of kindness can be seen as embedded in such processes, a response to the death of institutionally‐shaped solidarity, a way of isolated selves securing necessary acts of care and of feeling good about doing so, an ‘escape attempt’ (Cohen & Taylor, 1992) from uncertainty. Equally, however, kindness can be understood as evidencing, in the same uncertain context, that the lives of others do matter to us. Ultimately, kindness can be seen as both challenging and reinforcing of the relations that produce it in the first place. The interpersonal in Barbalet's account, then, is perhaps an unnecessarily diminished one, aligned with ego emotion, rather than as potentially world‐making and proto‐political. Kindness as interpersonal is neither about self *nor* other directed emotions: it is about both. Indeed, from this vantage point, the more relevant of Adam Smith's insights might be that the interests of self and others cannot be disentangled. Smith's writing also speaks to the complexity of emotions that surround the ‘pro‐social’, it is neither the ‘feeble spark’ of benevolence (137) nor self‐love that matters he argues but the ‘stronger love’—the love of feeling ourselves honourable and, as Debes (2015) puts it, ‘worthy to be cared about’. The point is this: far from indicating a restricted range of emotions, investigating attachment to kindness points to the sheer complexity and breadth of contemporary emotional life. In what follows, the large‐scale historical shifts described by Barbalet can be seen as the background against which the contemporary turn to kindness happens, but I also argue for a focus on attachment happening through the everyday interpersonal—an uncertain,^23^ ambivalent space, both insignificant and significant, restricted and world‐making.

## 10 ATTACHING THROUGH CULTURAL OBJECTS, PRACTICES AND EMOTIONS

How, in practice, does this contemporary attachment to kindness unfold? Griswold (1986: 5) understood cultural objects as ‘shared significance embodied in form’ and the discussion of the kindness industry above is consistent with ideas about kindness being considered as cultural objects, materialised in a wide range of ways, from days of celebration to workplace performance indicators.

Such objects vary in their power, by which Griswold (1986) means their capacity to shape beliefs and behaviours. Drawing on the notion of ‘fit making’, Hallett et al. (2019) write that the risk of circularity—that an idea fits because it is resonant or it is resonant because it fits—can be broken by seeing resonance as emergent, something that develops out of an attempt to *resolve* something (see also McDonnell, 2023). This is not dissimilar to the idea of enchantment as problem solving that I will investigate further below.

Attachment to, and through, ideas happens when there is a sweet spot between a sense of familiarity and novelty (McDonnell et al., 2017). RAK, I suggest, achieved this Goldilocks position: unusual or quirky enough to attract attention and to be legitimised by institutions but not so ‘out there’ that it risked rejection. Part of this legitimation, as I have shown, happens through the academy. Here, Cheng et al. (2023) argue, we need to look beyond the role of ‘champions or star entrepreneurs’ to already *established* bodies of knowledge. In the context of kindness, I have shown how contemporary understandings have become boot‐strapped to an established body of positive psychology knowledge that has grown up around happiness.

But of course we cannot understand contemporary attachment to the idea of kindness purely in terms of how it has been promoted or theorised academically. Indeed, the growth of interest in kindness within academia appeared to *follow* the wider cultural attachment to RAK and the idea of kindness more generally. That wider attachment is also rooted in the significance of everyday practices. Influenced by Actor Network Theory, Hennion has written extensively on the role of *practices* in relation to attachment through empirical studies of taste but also of care and addiction (Hennion & Vidal‐Naquet, 2017; Pomiès & Hennion, 2020). Kindness is not only an idea or a cultural object, then, but—like care—a practice, and there is plenty of empirical evidence that, in everyday life, ‘small acts of kindness’ are associated with making a material difference (Brownlie & Anderson, 2017; Ferguson, 2016).

There are different dimensions to how attachment unfolds through practices, including through collectives and techniques (Pomiès & Hennion, 2020). Practices that come to be called kindness *constitute* collectives. The idea of the collective resonates with what has been called the infrastructural nature of kindness—the complex web of social relations that enable ‘kindness’ but which ‘kindness’ in turn constitutes (Brownlie & Anderson, 2017).

Hennion's concept of ‘equipment’—the means of sustaining these collectives—is akin to what in research on kindness have been called ‘materialities of care’ (Buse et al., 2018). Again, however, these background materialities—such as the architecture of our neighbourhoods—are not passive: they are doing things for, and to, people in particular social contexts and at particular times.

In such theorising on attachment—whether through ideas or practices—emotions tend to come in and out of view, and explicit engagement with them is rare. Yet the academic words that have come to be associated with attachment (such as affinity and allegiance) as well as lay descriptions (“feeling a weight”, “being shaken”, Felski, 2020) speak to emotional impact.

Some theorists working on attachment certainly want to avoid attachment being reduced *only* to emotion. Felski, for instance, is clear that attachments are forged and not just felt. Yet there is not much of a sense in her work of emotions as themselves forged, that is as socially and historically shaped. Felski writes of a fear that the language of attachment ‘is a brief for mawkish outbursts’. She seeks to dispel this anxiety by making clear that attachment is not just about ‘warm and fuzzy feelings’ but inclusive of irony, presumably a more acceptable state. But why emotions are reduced to ahistorical fuzzy feelings in the first place is less clear.

Emotions *do* appear as more complex, embodied and interpersonal in Hennion's work on care and constraint but, again, *how* emotions shape attachments in specific socio‐historical and cultural contexts such as those described above is not made explicit in his work either (Hennion & Vidal‐Naquet, 2017). One obvious explanation for this lack of engagement with emotion is the influence of Actor Network Theory. Writing from an ANT perspective, de Laet et al. (2021, p. 810) note that ‘attachments should not be confused with affects’ where affect is understood as a ‘psychological force’. In the process, however, it seems that emotions as a *sociological* force have remained under‐theorised. There are cultural sociologists and theorists who have been more explicit about emotion in relation to attachment to cultural objects—Ahmed (2010) and Illouz (2018), for instance, have both written about attachment to happiness—but less about *how* this happens through everyday relationships and materialities. In the remainder of the article, I seek to understand sociologically the role of emotion in attachment to the idea of kindness at this particular juncture through focussing on kindness as everyday enchantment.

## 11 MAKING CURRENT TIMES BEARABLE: KINDNESS AS EVERYDAY (AGENTIC) ENCHANTMENT

I have been on the receiving end of so much kindness and thoughtfulness. Our lovely neighbours shopped for us while we were isolating after Mum's death. Friends have left cake and gin on the doorstep. Cards, flowers and letters have cheered me up with memories of my parents. These small acts have made a big difference. Never despise these little things, or think them insignificant. They are the best of us and they are what make our current times bearable.

This extract from an article written by journalist Cathy Killick (2021) in the aftermath of losing both her parents to Covid‐19 gives a sense, despite the specificity of the context, of how kindness is talked about in current times. Contemporary everyday accounts of kindness certainly vary by social circumstance but they are often, as in this case, about mundane things—cards, cakes and letters—small interactions which, nevertheless, enable fundamental things to happen. Research on acts that are associated with kindness resonate with Killick's account and emphasise the material difference that such acts can make to everyday lives, especially in the context of growing socio‐economic pressures.

Kindness, then, comes to matter to people in part because of these direct material effects, but also through the everyday narration of such acts, in which two aspects in particular have taken hold: the notion of something big/transformative happening through something small/ordinary; and of kindness as happening unexpectedly, ‘as if by magic’, or at least fleetingly, contingently and not out of obligation. These features—transformation, unexpectedness^24^ and a perceived lack of obligation—are core to what makes contemporary kindness feel enchanting and hence, I argue, to why it is attached to especially strongly at this particular time. Evidence for the resonance of these characteristics in everyday lives, but also for the ambivalence felt about the idea of kindness, can be found in empirical research on kindness in recent years across a range of settings, including communities (Brownlie & Anderson, 2017; Ferguson, 2016); education (Back, 2016; Burton, 2021), health (Vanhaecht, et al., 2020) and refugeedom (Smith, 2020). This research often shares with the Kindness Industry content a focus on small, supererogatory acts but, unlike this content, it reveals these acts as socially embedded and as enchanting *and* ambivalent.^25^

After Morgan (2018), enchantment can be understood as involving a sense of things as outside our control because of being ‘at the hands of a device or feeling or experience that places one in the service of another’. Though such lack of control may seem unappealing, we often seek out this disruption to our everyday lives. Usually framed as happening when we seemingly cannot explain a phenomenon—love for instance—this understanding wrongly implies that when an explanation is found, enchantment will be abandoned. Instead, Morgan (2018) suggests the problem‐solving nature of enchantment means we continue to invest in it as long as it meets our needs. This is more than wish fulfilment as it is also about ‘the things we do and how we do them to make the world go our way’. Morgan's focus on enchantment as an agentic transformative practice that is *felt* as beyond our control is, I argue, at the heart of attachment to kindness.

These problem‐solving qualities of enchantment, however, are historically shaped. Ideas of kindness as enchanting, for example, were clearly intensified during the recent pandemic when kindness assumed talismatic qualities that could ward off danger, leading people to feel protected but also to act in protective ways^26^—but they are also amplified in the context of the wider global shifts described by Barbalet.

Morgan's framing of enchantment is a productive one but I do not follow him in his separation of the emotional from the rational. Instead, emotions in the context of enchantment, as in the rest of social life, are the very means through which the complexities of the world are navigated (Brownlie, 2014; Holmes, 2010). We can see this happening in the framing of contemporary kindness as enchanting, particularly in relation to feelings of hopelessness and shame. These emotions are intensified in the face of increasingly global problems and of ‘monolithic social forms’ (Featherstone, 2020)—problems that individual states cannot or will not act to address—*and* by the individualisation of responsibility for one's wellbeing that is core to neoliberalism.

In this context, framing small acts of kindness as transformational offers hope to individuals (who are ‘being kind’) that they can make a difference at a time when feelings of powerlessness are acute. The naming of kindness as a means of resistance to divisive populism in the US through the ‘Make America kind again’ slogan, for instance, resonated not just because it is more accessible than say ‘Make America fraternal (or compassionate) again’—but because the idea of ‘kindness’ speaks to the possibility of people being able to achieve social change, cumulatively, through ‘ordinary’ (yet transformative) small interpersonal acts, even in the face of seemingly unstoppable broader developments. At the same time as allowing for a greater sense of agency,^27^ kindness also *feels* enchanting, because it is facilitates Smith's ‘stronger love’, that is, self‐approbation or the approval of self (Hanley, 2009).

There is an emotional basis, then, to why kindness seems enchanting for those offering it but understanding kindness as enchanting, as happening unexpectedly and/or as unobligated, also achieves something for those on the receiving end. In particular, it offers a way around what Mauss (1924) calls ‘the wounding’ of being offered help, specifically diluting feelings of shame, dependency and vulnerability. In other words, those on the receiving end of kindness are able to frame it as others giving when they did not need to, even if those offering the help may experience their actions as something closer to duty or obligation. Placing the onus on the giver's lack of obligation rather than the receiver's needs (and perhaps also demands) allows the difficulty of being helped, of being recognised as dependent, to be almost magically diluted or reframed even as the inequalities in *who* is giving (or receiving) are left unexamined. This emotional work is necessary because a sense of depletion, of being undone by kindness or of feeling shame about it, is a part of how receiving kindness is currently experienced not just in Britain but in other cultures too (Cole, 2019; Ferguson, 2016). Salmela (2019) writes of shame as *the* master emotion of neoliberalism, operating at both an interpersonal and collective level, though its roots go much deeper (Hoggett et al., 2013). These moral scripts are socially and culturally shaped—including through political discourses that emphasise the moral worth of those deemed active and the lack of value attached to those positioned as inactive—and are echoed in the accounts of those experiencing kindness (Brownlie & Anderson, 2017).

Emotions are also core to the understanding of kindness as enchanting because the sense of it as unexpected, as apparently not *having* to happen, makes the gratitude felt towards the person offering kindness all the more intense. Gratitude and love have long been associated with the notion of going ‘over and above’ (Cantó‐Milà, 2013; Simmel, 1964). To construe kindness as enchanting, then, is based on the idea that we do things without having to—the converse, then, of Bottero's (2023) ‘grudging acts’, those things we feel we have to do despite not wanting to. Nearly a decade ago, Iorio (2014) argued that sociology required a new concept to understand this ‘extra’ quality. He looked to Boltanski's (1990) reworking of the Christian concept of agape. Agape, Iorio (2014) argued, involves overabundance in the sense that it offers more than the situation demands and, unlike exchange‐based ideas (including ‘the gift’), does not presuppose reciprocity or conditionality.^28^ In a wide range of contemporary stories of kindness what is often being imagined is exactly this—that people are acting in unexpected and unobligated ways. Kindness comes to *feel* ^29^ enchanting then because of delight at ordinary but also transformative and surprising encounters in the everyday.^30^ In the contemporary kindness industry, too, as we have seen, these stories are commonplace but often take the form not just of contingency and unexpectedness but of randomness.

The framing of kindness as enchanting potentially addresses, then, feelings of hopelessness and of shame but never completely, as kindness, in the end, remains highly selective. Like all enchantments, it does not exist outside of power relations and inequalities (Ham & Sunuwar, 2020). To attach to the idea of kindness is to live with this potential instability, the potential of enchantment and the concomitant risk of disenchantment. Rather than sit with this instability and ambivalence, however, recent social theoretical accounts of kindness have tended either to over‐invest in kindness as radical and utopic (Baccolini, 2017) *or* fallen into the kind of despising, or at least dismissive, tone Killick warns against and which we can hear, even if unintentionally, in both Cazenave and Illouz's accounts of everyday emotional investments in the context of capitalism.

Knowledge of disenchantment, as Morgan has argued, does not mean we necessarily stop investing in the enchanting aspects of life—and indeed these persist as a way of coping with the emotional fallout of rationalisation. Resonating with work which sees enchantment both as a tool for the development of markets *and* as beyond complete calculation (Campbell, 1987)^31^—in other words, enchantment as *both* creative agency and rationalised manipulation (Beckert & Bronk, 2018)—this sense of messy ambivalence, of the relationship between enchantment *and* disenchantment, imaginative agency *and* regulation, needs engaged with in theoretical work on the commodification of emotion and, in particular, emotions associated with the ‘pro‐social’.

## 12 CONCLUSION

The idea of kindness has come to matter—or taken a hold—in many contemporary English‐speaking societies with a striking intensity and in particular ways. My aim has been to offer an understanding of this preoccupation beyond seeing it only as part of a wider process of commodification of the ‘pro‐social’. I illustrated this wider process through outlining the rise of RAK and other cultural objects associated with kindness and describing the industry that has grown up around them. I suggested, in effect, that the capitalist large‐scale processes and ‘cultural scenarios that we did not write’ (Illouz, 1997, p. 293) are certainly the waters being swum in, but that those doing the swimming, what they feel and do, need greater attention paid to them. This involved looking at how and why the idea of kindness is both more actively engaged with, but also more ambivalent, than the simple framing of it as an emodity.

To do this work I drew on theorising on attachment and enchantment, and on sociological work on emotions and relationships. This is because there is an inescapably *felt* quality to how, and why, we attach the idea of kindness to particular acts. There is nothing intrinsic to the acts themselves that makes them kind.

Research on ‘everyday kindness’ has surfaced the extent to which—especially in the deeply individualising conditions of contemporary capitalism—kindness is also understood as making a material difference to everyday lives; as Killick put it, to making life bearable. This happens ambivalently as kindness relates to both joy and shame, to the apparently insignificant and the consequential. Enchanting ideas of transformation, of unexpectedness and lack of obligation may be fantastical in the face of evidence of how social context *actually* shapes such acts, but they do offer ways of managing ambivalent, difficult emotions arising from contemporary socio‐political circumstances—not least hopelessness, *as well as* the challenging emotions associated with kindness itself, including shame. Ultimately, then, it is not just the material consequences of the idea of kindness that make it worth investing in, though these are hugely important in their own right (Anderson et al., 2015), but also the problem‐solving emotional consequences of viewing kindness as enchanting.

In the context of deepening social divisions, recent theorising about collective emotions has tended to focus on social movements and populist uprisings (Salmela & von Scheve, 2017). But ‘quieter’ solidarities were highlighted during the pandemic when webs of everyday relationships came to be valued, albeit unequally (Andersen et al., 2022); and this world‐making *and* enchanting potential of the interpersonal has long been recognised in sociology, most notably through feminist writing (Federici, 2018). Against that backdrop, this article can be read as a general call to keep, alongside other relations, the interpersonal (and its political consequences) in our theoretical sights. More specifically, it has been an attempt to (re)focus on the ambivalences of contemporary capitalism and the ways in which these play out through our experiences of attachment, enchantment and how others come to matter to us—to put it another way, on how it is that we stay afloat in the waters we all have to swim in.

## Data Availability

The data that support the findings of this study are available from the corresponding author upon reasonable request.

## ENDNOTES

^1^
Random Acts of Kindness Foundation, ‘‘The History of Random Acts of Kindness Day ‐ February 17th’, The Kindness Blog [web blog], https://www.randomactsofkindness.org/the‐kindness‐blog/5‐the‐history‐of‐random‐acts‐of‐kindness‐day‐february‐17th (accessed 3 June 2024).

^2^
Improvised Life [web blog], ‘The Origins Of ‘Random Kindness And Senseless Acts Of Beauty’’ https://improvisedlife.com/2013/06/11/the‐origins‐of‐random‐kindness‐and‐senselss‐acts‐of‐beauty/ (accessed 3 June 2024).

^3^
The English speaking world includes a disparate range of countries, but there is evidence that discourses of kindness as small ‘good’ acts that ‘make a difference’ are being circulated in India, Singapore and New Zealand as well as in the Global North (Dutta & Elers, 2020).

^4^
See Jackson's 1993 article ‘Even sociologists fall in love’.

^5^
C.W. Mills (2002) referred to unquestioning employees of 1950s America as ‘cheerful robots’.

^6^
See https://www.theworldkindnessmovement.org/members/ (accessed 3 June 2024).

^7^
For US organisations, see https://charitycheck101.org/results/. For the UK, see https://register‐of‐charities.charitycommission.gov.uk/charity‐search. (accessed 3 June 2024).

^8^

https://www.kindspring.org/ (accessed 3 June 2024).

^9^

https://kindness1billion.org/ (accessed 3 June 2024).

^10^
I draw here on Green and Moran’s (2021: 172) description of scientisation as ‘the reification and normalisation of scientific language, knowledge and behaviours’.

^11^
Cultural representations of kindness, although an important part of the ‘industry’, are not the focus of this article.

^12^
The overall field of academic publishing increased over the past 20 years, but was still outpaced by the increasing volume of publications about kindness. Between 2000 and 2022 we see a 3‐fold increase in the volume of total academic publications (from 1 m publications in 2000 to 3.1 m in 2022), during which time the volume of publications about kindness increased almost 5‐fold (from 17 publications in 2000 to 83 in 2022).

^13^
For a recent example see BBC Radio 4 ‐ Just One Thing ‐ with Michael Mosley, Be Kind (2024).

^14^
The Sussex Centre for Research on Kindness, for instance, which receives much of its funding from Kindness UK, collaborated with the BBC broadcaster, Claudia Hammond, now visiting professor at Sussex University, to undertake a large‐scale online survey linked to a series of programmes on BBC Radio 4, ‘the Kindness Test’. Hammond subsequently published her own contribution to the burgeoning ‘popular kindness’ literature entitled The Keys to Kindness: How to be Kinder to Yourself, Others and the World (Hammond, 2022).

^15^

https://thekindnesscorporation.com/ (accessed 4 June 2024).

^16^
See https://study‐online.sussex.ac.uk/online‐courses/psychology‐of‐kindness (accessed 4 June 2024).

^17^
See, for example, https://talkedaboutmarketing.com/blog/kindness‐in‐business‐a‐pathway‐to‐profit‐and‐happiness/ (accessed 4 June 2024).

^18^
Asda Café celebrates Random Acts of Kindness Day® https://corporate.asda.com/newsroom/2022/02/15/asda‐cafe‐celebrates‐random‐acts‐of‐kindness‐day (accessed 4 June 2024).

^19^
Leading with kindness: A report on the learning from the Kindness Leadership Network (Carnegie UK Trust, 2021).

^20^
From a different perspective, Wilkinson (2018) notes Elias' (1994) argument that rationalization and a preoccupation with compassion can co‐exist.

^21^
In recent work Illouz appears to offer greater credence to everyday understandings, drawing the distinction between flawed and false ideology in order to not ‘dismiss and negate the thinking and feeling of citizens’ (2023:7)

^22^
This is not an entirely new claim. Cohen and Taylor (1992), drawing on Lasch's (1985) idea of a ‘minimal self’, noted that ‘faced by external dangers and diminution of political hopes, we settle for psychic survival’

^23^
Kindness, like the interpersonal trust mentioned by Barbalet, happens in circumstances of uncertainty—if the situation was completely predictable, trust would not be necessary and ‘kindness’ (at least as it is currently framed as above and beyond) would not happen.

^24^
To frame kindness as enchanting in part because it is unexpected is not to suggest kindness is always a surprise but it is to argue that in contemporary times, if everyday acts of help and support become routinised and obligated, they will not necessarily be named, or felt, as kindness.

^25^
Evidencing the impact on everyday lives of discourses such as those from the ‘kindness industry’ is not straightforward. Injunctions to be kind, as we saw during the recent pandemic, can be experienced as regulatory and prescriptive but also welcomed by some as solidaristic. Recent signs of a kindness ‘backlash’ in some sectors (McCartney, 2023) also points to the instability of such discourses over time.

^26^
For an example of this see Albuquerque (2020).

^27^
This touches on the strength of kindness relative to *some*, restricted, versions of justice. See, for instance, Adam Smith's (1984) observation that ‘we may often fulfil all the rules of justice by sitting still and doing nothing’.

^28^
Mauss (1924) refers to Malinowski's (2014) category of the ‘pure gift’ as capturing a similar absence of reciprocity though Mauss himself thought this classification ‘inapplicable’.

^29^
See also Bennett (2001) and Bhatti et al. (2008) for an account of how enchantment *feels*, the latter focussing on the emotional power of unexpected transformation; the disenchanted world, in contrast, determined by calculation.

^30^
Scott (2021) defines surprise as a reaction to the sudden discovery of unexpected knowledge and makes the point that it feels less morally charged than embarrassment because it is seen as beyond our control.

^31^
Weber, of course, remains a touchstone for those seeking to understand enchantment in the context of capitalism (see Ritzer, 1999) and while we need to be cautious of reading these as private relationships (Symonds & Pudsey, 2007) it is interesting that it is in the ‘direct and human relations’, ‘in pianissimo’, that Weber found the residues of the ‘enchanted’ world (1948: 155)
